# Unlearning versus savings in visuomotor adaptation: comparing effects of washout, passage of time, and removal of errors on motor memory

**DOI:** 10.3389/fnhum.2013.00307

**Published:** 2013-06-28

**Authors:** Tomoko Kitago, Sophia L. Ryan, Pietro Mazzoni, John W. Krakauer, Adrian M. Haith

**Affiliations:** ^1^Motor Performance Laboratory, Department of Neurology, Columbia University College of Physicians and SurgeonsNew York, NY, USA; ^2^Department of Neurology, Johns Hopkins UniversityBaltimore, MD, USA; ^3^Department of Neuroscience, Johns Hopkins UniversityBaltimore, MD, USA

**Keywords:** adaptation, visuomotor rotation, unlearning, decay, savings

## Abstract

Humans are able to rapidly adapt their movements when a visuomotor or other systematic perturbation is imposed. However, the adaptation is forgotten or unlearned equally rapidly once the perturbation is removed. The ultimate cause of this unlearning remains poorly understood. Unlearning is often considered to be a passive process due to inability to retain an internal model. However, we have recently suggested that it may instead be a process of reversion to habit, without necessarily any forgetting *per se*. We compared the timecourse and nature of unlearning across a variety of protocols where unlearning is known to occur: error-clamp trials, removal of visual feedback, removal of the perturbation, or simply a period of inactivity. We found that, in agreement with mathematical models, there was no significant difference in the rate of decay between subject who experienced zero-error clamp trials, and subjects who made movements with no visual feedback. Time alone did lead to partial unlearning (over the duration we tested), but the amount of unlearning was inconsistent across subjects. Upon re-exposure to the same perturbation, subjects who unlearned through time or by reverting to veridical feedback exhibited savings. By contrast, no savings was observed in subjects who unlearned by having visual feedback removed or by being placed in a series of error-clamp trials. Thus although these various forms of unlearning can all revert subjects back to baseline behavior, they have markedly different effects on whether long-term memory for the adaptation is spared or is also unlearned. On the basis of these and previous findings, we suggest that unlearning is not due to passive forgetting of an internal model, but is instead an active process whereby adapted behavior gradually reverts to baseline habits.

## Introduction

Human subjects adapt rapidly to systematic perturbations to their movements through an error-driven, model-based learning mechanism (Huang et al., 2011; Haith and Krakauer, 2013). However, behavior rapidly reverts to baseline when the errors that drive adaptation are removed. Although behavior in adaptation paradigms has been studied in tremendous detail, this process whereby recent adaptation is apparently forgotten remains poorly understood. We will adopt the term *unlearning* for the reversion to baseline. We do so because it allows us to remain agnostic as to whether reversion to baseline reflects decay (forgetting), or competition between intact memories.

Unlearning of a perturbation can occur in at least four distinct ways. Switching off the perturbation leads to errors in the opposite direction to those which drove the initial adaptation, leading to rapid adaptation back to baseline. However, unlearning can also occur in more spontaneous fashion if movement errors are artificially eliminated through error-clamp paradigms that create the illusion of perfect performance (Scheidt et al., 2000; Criscimagna-Hemminger and Shadmehr, 2008; Huang et al., 2011; Shmuelof et al., 2012). For purely visual perturbations, errors can be removed entirely by removing visual feedback, which also leads to a steady return toward baseline (Galea et al., 2011). Finally, unlearning can simply occur with the passage of time; sitting idle for a period of minutes to hours leads to a reduction in the extent of compensation for a perturbation (Criscimagna-Hemminger and Shadmehr, 2008). All of these manipulations lead to ostensibly the same outcome: that subjects make movements that are the same as those made at baseline. However, just because all four conditions lead to a reversion to the same baseline phenotype does not mean that they are in the same state in terms of retained motor memories (Smith et al., 2006).

Adaptation is commonly described mathematically with the state space model framework (Thoroughman and Shadmehr, 2000; Donchin et al., 2003; Cheng and Sabes, 2006; Zarahn et al., 2008). This framework essentially assumes that subjects adapt their behavior in proportion to the size of performance errors. The same set of equations describing learning can be derived based on assumptions of gradient descent on the squared movement error, or based on Bayesian estimation of the imposed perturbation. Unlearning can be conveniently accommodated in such models through a trial-to-trial forgetting rate. This forgetting rate also has the benefit of being able to capture the fact that adaptation is never able to quite reach an asymptote of zero error; learning from residual error in each trial is eventually balanced by unlearning between trials. Although all four varieties of unlearning described above can be modeled within the state space model framework, simply describing the data mathematically overlooks the deeper question of why unlearning should occur at all.

Adaptation according to state space dynamics is generally thought to occur through updating of an internal model that predicts the outcomes of a motor command. However, we and others have recently shown that an additional success-based, model-free learning mechanism (Huang et al., 2011; Izawa and Shadmehr, 2011) also plays a role in adaptation. In particular, the phenomenon of savings, i.e., faster re-learning upon re-exposure to a previously encountered perturbation, depends on this model-free learning mechanism (Huang et al., 2011). The fact that both model-based and model-free learning processes participate during adaptation raises the question as to which of these processes actually gives rise to the unlearning. In this study, we compared four different methods of eliciting unlearning: error clamps, removal of visual feedback, washout by removal of the perturbation, and the passage of time. We hypothesized that these four different manipulations would result in qualitatively different kinds of unlearning that would be revealed both by the time-course of the unlearning itself and by the presence or absence of savings on subsequent re-exposure to the original perturbation.

## Materials and methods

### Experimental methods

Forty healthy, right-handed individuals (age 28.3 ± 7.4 years, 18 women) were recruited from the local community. All participants were naïve to the purpose of the study and signed a written consent form that was approved by the Columbia University institutional human research review board.

**Figure 1:**
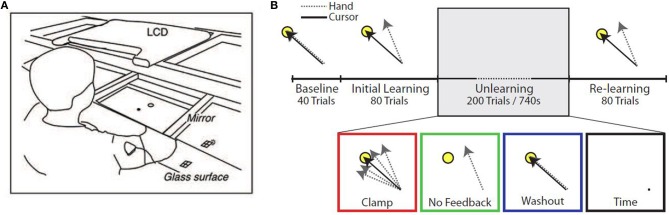
**Experimental setup. (A)** Diagram of experimental apparatus. **(B)** Experimental protocol. Subjects performed up to 400 trials in a single block, divided into four epochs: *Baseline* (veridical feedback), *Initial Learning* (30° counterclockwise rotation), *Unlearning*, and *Re-learning* (30° counterclockwise rotation). During the *Unlearning* epoch, subjects experienced one of four manipulations: task error clamped to zero (Clamp), removal of visual feedback (No Feedback), veridical feedback of hand position (Washout), or inactivity for 740 s (Time).

Subjects were seated at a glass surface table and moved a cursor by making planar reaching movements (Figure 1A). Hand position, calibrated to the position of the fingertip, was monitored using a Flock of Birds (Ascension Technology, Burlington, Vermont, USA) magnetic movement recording system at a frequency of 120 Hz. Real-time hand position was used to control the visual display and to provide on-line visual feedback. The hand itself was not visible to subjects. One condition of the experiment made use of error-clamp trials, in which the angular position of the cursor relative to the start location was clamped to a straight line between the start location and the target. Subjects still maintained direct control of the radial distance of the cursor from the start location during these error-clamp trials.

Subjects were instructed to make out-and-back movements from a center start circle to a single target (radius 1 cm, at the 135° position, 8 cm from the start circle), reversing within the target. The experimental paradigm consisted of 4 epochs (Figure 1B). The first epoch (*Baseline*) consisted of 40 trials with unperturbed feedback. The second epoch (*Initial learning*) consisted of 80 trials in which visual feedback was rotated 30° counterclockwise (CCW). In the third epoch (*Unlearning*), subjects were placed in one of four unlearning conditions: (1) 200 error-clamp trials (*Clamp*), (2) 200 trials with no visual feedback (*No Feedback*), (3) 200 trials with veridical visual feedback (*Washout*), or (4) sitting idle for 740 s (~12 min) (*Time*), which was the average amount of time taken by subjects in the other groups to complete 200 trials. In the final epoch (*Re-learning*), subjects were re-exposed to the perturbation for a further 80 trials to test whether any memory of the prior adaptation would be present in the form of savings.

### Data analysis

Trajectory data were smoothed using a 2^nd^-order Savitzky–Golay filter. Movement initiation was determined based on the first time that movement speed exceeded 2.4 cms^−1^. Initial reach direction was determined based on the angle between lines connecting the hand position at movement initiation with position of the hand at peak velocity and the center of the target. We subtracted from this reach angle a baseline reach direction for each subject, estimated from the last 20 trials of the *Baseline* epoch.

We determined the rate of unlearning for each subject through the slope of a linear regression between the initial reach directions on consecutive trials over the course of the *Unlearning* epoch. We quantified the overall extent of decay in the *Unlearning* epoch by taking the ratio between the reach direction immediately preceding (last 20 trials of the first adaptation block) and following (first trial of the second adaptation block) the unlearning block.

To assess savings, we assumed that subject behavior followed a linear state-space model given by:

(1) $$x_{i+1}=Ax_{i}+Be_{i}+\eta_{i}$$

(2) $$y_{i}=Cx_{i}+\varepsilon_{i}$$

In this model, *x*_i_ corresponds to the state of the subject's internal model of the perturbation on trial *i, y_i_* reflects the hand position on trial *i*, and *e_i_* represents the directional error on trial *i*. *A* ≤ 1 is the trial-to-trial retention rate, *B* is the adaptation rate, *C* = 1, and η_*i*_ and ε_*i*_ are independent noise terms, with η_*i*_ : *N*(0, *Q*) and ε_*i*_ : *N*(0, *R*), and *x*_0_ : *N*(μ, *V*_0_). We estimated the remaining parameters (*A, B*, μ, *V*_0_, *Q, R*) separately for each individual subject using maximum likelihood estimation (Ghahramani and Hinton, 1996; Cheng and Sabes, 2006). Trials that were excluded were treated as unobserved variables by setting *C* = 0 on these trials. In order to minimize the risk of overfitting the model by allowing too many free parameters, all parameters were assumed to be constant throughout the experiment except for the learning rate *B*, which we allowed. to take different values in each epoch. We considered savings to have occurred if the estimated value of *B* during re-learning was greater than the corresponding value during initial learning. A power analysis based on data from Zarahn et al. (2008) suggested that 9 subjects would be an appropriate minimum sample size using a power of 0.9 with two-tailed alpha of 0.05.

Note that we could, in principle, have allowed the forgetting rate *A* to also have varied across trials, since a change in *A* would also have influenced the learning rate. In practice, changing the forgetting rate A tends to have a far larger effect on the asymptote of learning than on the initial rate. Varying B has a strong effect on initial adaptation rate and a weaker effect on the asymptote. Although this means that these parameters can in principle be dissociated in the kind of data we consider here, in practice jointly estimating these two parameters from small datasets yields correlated estimates that are highly prone to overfitting (Cheng and Sabes, 2006). We therefore considered it best to compare estimated learning rates across epochs assuming all other things to be equal and therefore allowed only the learning rate *B* to vary across epochs.

## Results

Four groups of 10 subjects each participated in an experiment to test the effect of different types of feedback on prior visuomotor adaptation: *Clamp, No Feedback* (*NoFB*), *Washout* (*WO*), and *Time*. All groups exhibited a comparable amount of adaptation during the *Initial Learning* epoch. Across all subjects, the asymptotic error (last 20 trials of initial learning) was 7.4 ± 3.6°, and did not differ significantly across groups (*p* = 0.94).

### Influence of feedback type on unlearning

**Figure 2:**
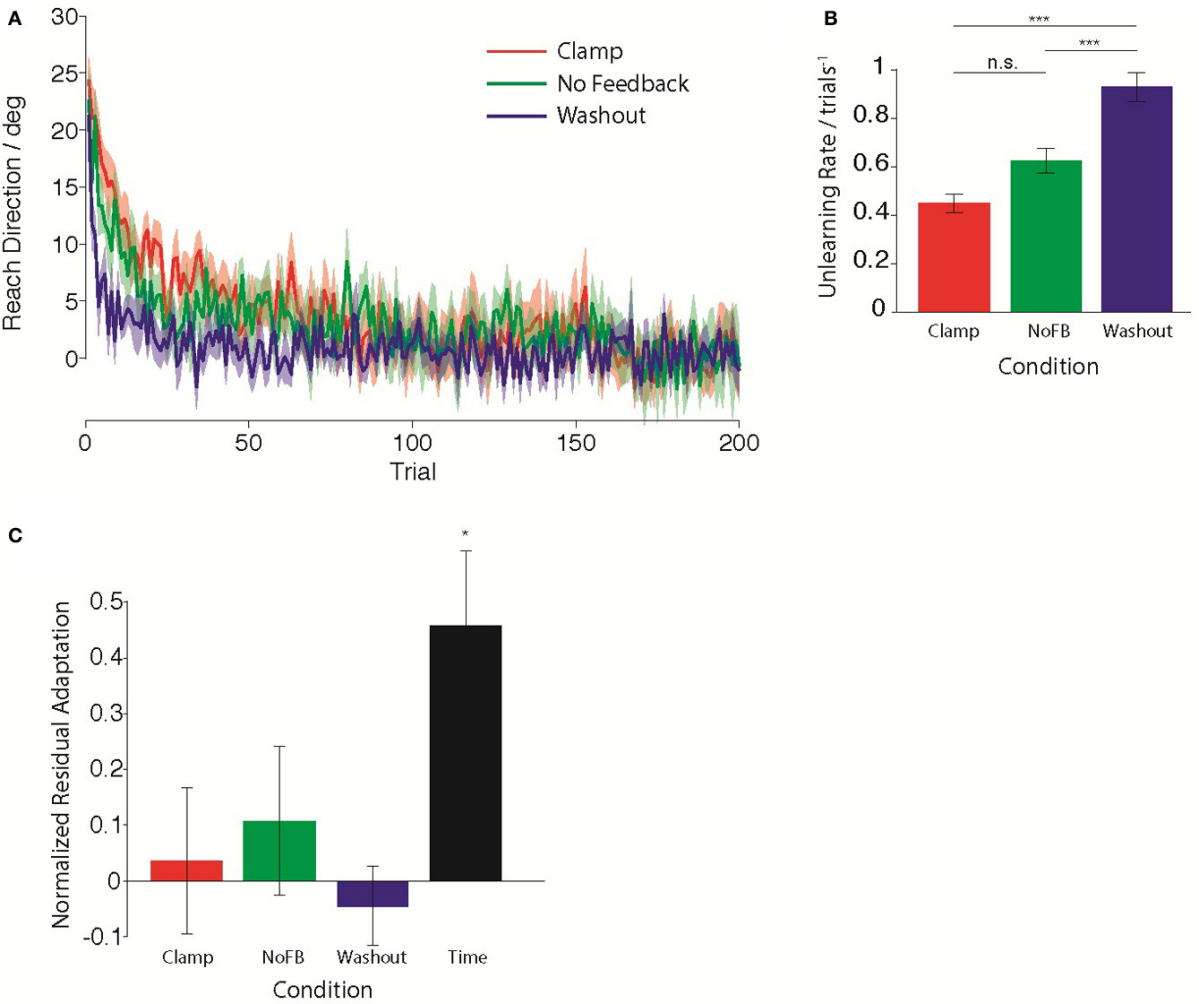
**Behavior during the *Unlearning* epoch. (A)** Mean timecourse of unlearning in the Clamp (red), No Feedback (green), and Washout (blue) groups. Zero reflects baseline behavior. Shaded error bars indicate standard error in the mean across subjects. **(B)** Mean unlearning decay rate. **(C)** Normalized residual adaptation, assessed as the difference between directional error on the first trial of the Re-learning epoch and the extent of initial learning (last 20 trials of second epoch), divided by the amount of initial learning. Asterisk indicates significant difference from zero.

First, we compared the trial-by-trial rate of unlearning in the *Clamp, NoFB*, and *Washout* groups. Figure 2A illustrates the average behavior for each group during the *Unlearning* epoch. Standard models of adaptation suggest that a constant proportion of prior adaptation is forgotten on each trial, leading to an exponential timecourse of decay. Assuming that this is the case, we estimated the time-constant of this unlearning by performing a linear regression between reach directions on consecutive trials. We quantified the decay rate as 1 minus the slope of the regression. For the *Clamp* and *NoFB* groups, this is equivalent to estimating *A* in Equation 1, for the *Washout* group this is equivalent to estimating *A* + *B* (although note that for the purpose of this analysis is was not necessary to fit a full state space model to the data). The estimated unlearning rates are shown in Figure 2B. The decay rate varied significantly across the three groups [*F*_(2, 27)_ = 37.8, *p* < 10^−7^]. As expected, there was a significant difference in the rate of unlearning between the *Washout* group and both the *Clamp* and *NoFB* groups [*t*_(9)_ = 7.86, *p* < 10^−4^; *t*_(9)_ = 5.84, *p* = 0.0012; Bonferroni-corrected). Although the rate of unlearning appeared slower in the *Clamp* group compared to the *NoFB* group, this difference was not statistically significant (*p* = 0.82 after Bonferroni-correction).

Next, we compared the total amount of retention of the initial adaptation by the end of the unlearning epoch. We determined the amount of retained adaptation for each subject through a retention factor that quantified the proportion of the total amount of initial adaptation that remained following the *Unlearning* block. Figure 2C shows the average retention factor across subjects for each group. Only the *Time* group exhibited retention that was significantly different from zero [*t*_(9)_ = −3.845, *p*_Time_ <0.05 Bonferroni-corrected; *p*_Clamp_ = 0.69; *p*_NoFB_ = 0.31; *p*_WO_ = 0.54), i.e., all other groups had returned to baseline. Although the *Time* group did not fully return to baseline, they did exhibit partial unlearning, evidenced by the fact that they had a retention factor that was significantly smaller than 1 [*t*_(9)_ = 3.414, *p* < 0.01).

### Presence of savings following the different unlearning protocols

**Figure 3:**
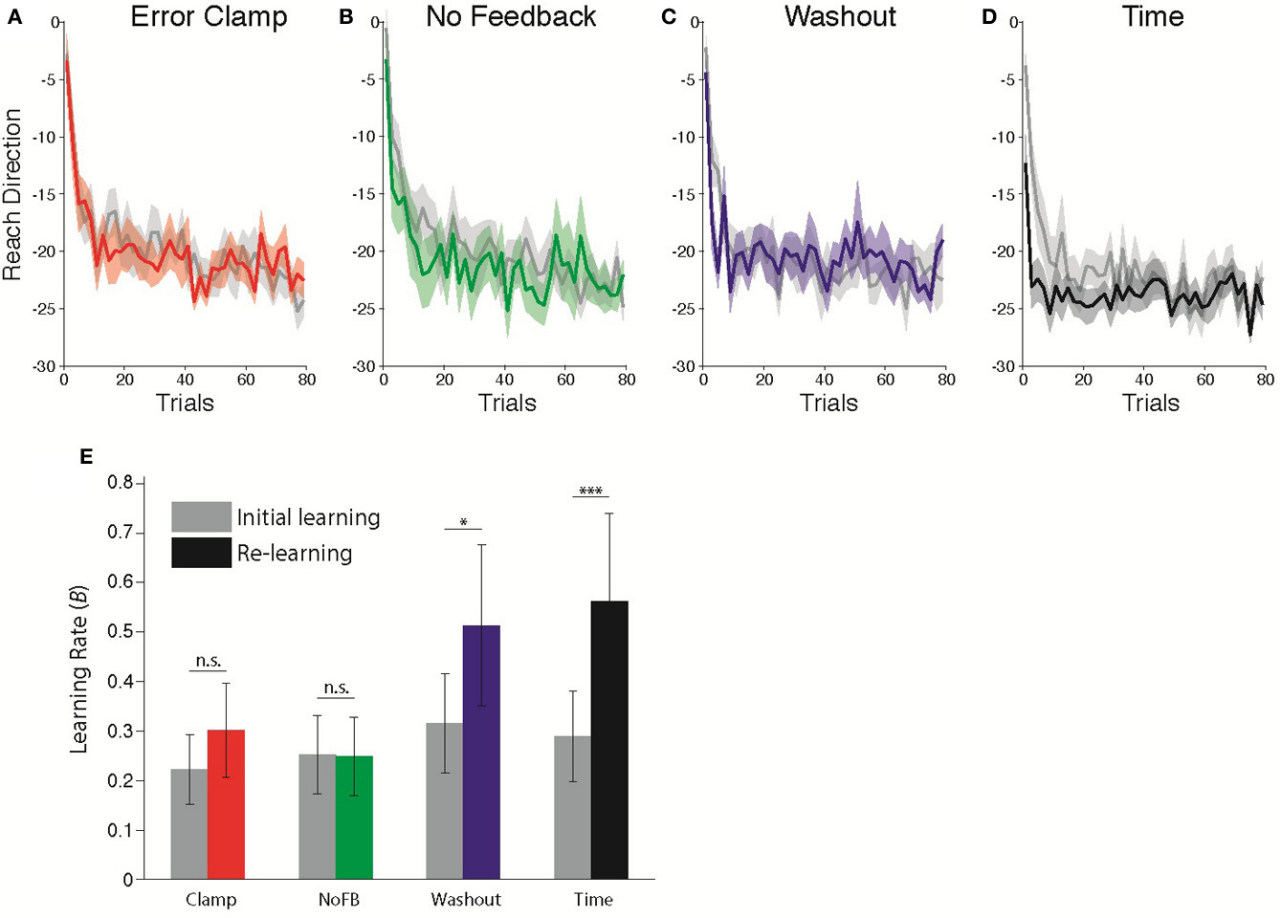
**Comparison of learning rates before and after *Unlearning*. (A–D)** Timecourse of *Initial Learning* (gray) and *Re-learning* (colored) for **(A)** Clamp, **(B)** No Feedback, **(C)** Washout, and **(D)** Time groups. Bins of 5 trials. Shaded error bars indicate standard error in the mean across subjects. **(E)** Estimated learning rates according to state space model fits (**B** parameter) during Initial Learning (gray) and Re-learning (color). Error bars indicate standard error in the mean across subjects. ^*^p = 0.0598, ^***^p < 0.001.

Following the unlearning manipulation, we re-exposed subjects to the 30° CCW rotation perturbation to assess whether or not a memory of the prior adaptation was present in the form of savings. Figures 3A–D compares the initial learning and re-learning.

Following (Cheng and Sabes, 2006; Zarahn et al., 2008), we fitted state space models to each subject's data (see Materials and Methods). The critical parameter of interest with regard to savings is the sensitivity to error, *B* in Equation 1. We allowed this parameter of the model to take different values in each epoch, in order to capture the difference in adaptation rates between the first and second exposures (Zarahn et al., 2008). All other parameters were assumed to be fixed throughout the experiment. Savings would therefore be evident as a change in the learning rate *B* during the *Re-learning* epoch relative to the *Initial Learning* epoch. Figure 3E shows the average estimated learning rate for each group during the *Initial Learning* and *Re-learning* epochs. We found that the change in learning rate was significantly different across groups (Mixed-effects ANOVA, Group × Epoch interaction, *F*_(3, 36)_ = 4.493; *p* < 0.01). *Post-hoc* comparisons showed a marginally significant change for the *Washout* group (*t* = 3.00, *p* = 0.0598, Bonferroni-corrected), and a strongly significant effect following time (*t* = 6.23, *p* < 0.001). Thus, we observed highly robust savings following unlearning due to the passage of time with no movements, less reliable savings following 200 trials of washout and no savings following either *Clamp* or *NoFB* blocks.

## Discussion

Trial-by-trial learning during adaptation paradigms is believed to depend on sensory prediction errors (Mazzoni and Krakauer, 2006) driving updates to an internal forward model in the cerebellum (Bastian, 2006; Tseng et al., 2007; Shadmehr and Krakauer, 2008; Taylor et al., 2010). This model of learning is expressed mathematically through state-space models (Thoroughman and Shadmehr, 2000; Donchin et al., 2003; Cheng and Sabes, 2006). Unlearning has typically been accommodated within such models through a trial-to-trial retention factor, with the general supposition that this unlearning reflects a forgetting of the recently-learned internal model.

The basic state-space framework may be extended to include multiple components that learn and decay at different rates (Smith et al., 2006; Körding et al., 2007). Enriching the model in this way enables it to account for the characteristic two-timescale learning curves, accounts for spontaneous recovery of recently washed out learning during clamp trials, and suggests a mechanism for savings: that the faster relearning is supported by a latent slow-process memory. However, such a model is unable to account for our results. The fact that learning passively returned to baseline during the *Clamp* and *NoFB* implies that even the slower-decaying process must have decayed back to baseline values. Although we saw no savings following this unlearning, consistent with the predictions of such models, we did observe savings following a comparable number of trials of washout by a null perturbation. This would not be predicted by a multi-rate state-space model, since the decay of the memory of the initial learning is governed purely by the number of trials since exposure and should therefore be the same as for the *Clamp* and *No Feedback* conditions. One way in which the state-space model may be extended in order to account for our findings would be to include a capacity to contextually switch between multiple learned states (Lee and Schweighofer, 2009; Berniker and Körding, 2011; Pekny et al., 2011). The difference between *Clamp*/*NoFB* and *Washout* could then be explained by the fact that washout trials did engage such a contextual switch, but *Clamp* and *NoFB* did not.

Our results demonstrate that removing errors altogether (*No Feedback*) has a qualitatively similar effect to artificially clamping errors to zero (*Clamp*), both in terms of the time-course of unlearning and the abolition of subsequent savings. The unlearning part of the result is predicted by the state-space framework since, in both cases, the only change in internal state between trials stems from the retention coefficient (*A* in Equation 1). The similarity between *Clamp* and *NoFB* is interesting because it suggests that in the absence of feedback, subjects may implicitly presume success based on their forward model predictions about the outcome of their movements. Sitting idle for a comparable passage of time had a far weaker effect of unlearning, implying that it is necessary to actively make movements in order for unlearning to occur.

There is a potential mechanism that may support a model-based interpretation of movement-dependent unlearning. Cerebellar learning depends critically on plasticity at the parallel fiber-Purkinje Cell (PF/PC) synapse (Coesmans et al., 2004; Jörntell and Hansel, 2006). Long-term depression (LTD) at this synapse occurs when simple spikes, movement related activity carried by mossy-fiber inputs to cerebellar cortex, co-occur with complex spikes, which are driven by climbing fiber inputs. This LTD must be balanced by long-term potentiation (LTP) in order for the cerebellum to be able to maintain flexibility in what it can learn. LTP occurs when simple spike activity occurs in the absence of complex spikes. Popular models of cerebellar learning posit that the climbing fiber signal reflects a prediction-error signal. The absence of a complex spike therefore should signal perfect performance. However, if no sensory feedback is available to validate the prediction made by the cerebellum, then presumably this must also be encoded by the absence of a complex spike. Thus, this mechanism can potentially explain both why making movements leads to strong forgetting (increased simple spike activity), while also explaining why unobservable errors should lead to similar amount of forgetting as observed zero error.

Although it may be possible to interpret our findings here in terms of multiple internal cerebellar-based internal models, our recent work has proposed a fundamentally different view of motor learning. We have argued that behavior in adaptation paradigms is in fact governed by a combination of two qualitatively distinct learning processes (Huang et al., 2011; Shmuelof et al., 2012; Haith and Krakauer, 2013). Although initial learning may proceed through updates to a forward model in a cerebellar-dependent, model-based manner consistent with state-space model dynamics, savings upon re-learning appears to be due instead to a distinct, model-free learning mechanism that depends on the basal ganglia rather than the cerebellum. Actions that prove to be successful during initial learning are remembered and recalled during subsequent exposures, leading to accelerated adaptation during re-learning (Huang et al., 2011). The slow process invoked by multi-rate state space models of learning may in fact inadvertantly provide a means to approximate model-free components of learning.

The presence of multiple, qualitatively different learning systems raises the question of which learning system the unlearning is truly occurring in. Specifically, unlearning might be a model-free phenomenon, reflecting a gradual reversion to old (baseline) habits, rather than forgetting of a forward model. It is quite possible that spontaneous unlearning in clamp trials and following removal of feedback is due to a combination of forgetting of a forward model and reversion to baseline habits. Indeed, unlearning behavior in clamp trials shows two distinct timescales (Smith et al., 2006), suggesting that two distinct processes are implicated.

We recently showed that the point which subjects decay to in clamp trials can be shifted to a new action by inserting a period of binary reinforcement of an adapted action (Shmuelof et al., 2012). Vector error feedback about task performance was removed following initial adaptation, forcing subjects to rely on binary feedback alone and precluding them from maintaining and using an accurate internal model. Thus, subjects had to rely on an alternative learning strategy, which we hypothesize uses the same model-free mechanism that is responsible for savings (Huang et al., 2011). This result can be explained quite naturally within a multiple learning systems framework in terms of a shift in the balance between learning systems caused by the removal of vector error. However, it is problematic to explain this result within a state-space model framework in which learning of all components is driven by vector error.

The fact that changing the nature of feedback can alter patterns of unlearning suggests that unlearning may usually occur because of a reversion to a baseline, model-free habit, rather than as a consequence of passive unlearning of an internal model. A partial reduction in the amount of decay is also seen following transcranial direct current stimulation of the motor cortex (Galea et al., 2011). We similarly interpret this result as being due to the promotion of model-free learning in motor cortex and not to halting decay of an internal model. Interestingly, transcranial direct current stimulation of the cerebellum accelerates initial adaptation but has no effect on the timecourse of unlearning (Galea et al., 2011), further calling into question the notion that unlearning is a cerebellar-based phenomenon. Although these previous experiments suggest that unlearning is due to an active return to a habitual baseline rather than passive decay of a recently-learned internal model, this does not necessarily mean that the forward model is not also forgotten. It is difficult to establish the state of the internal model when overt behavior may be dictated by additional overlying processes.

Unlearning during washout was faster than in the *Clamp* and *NoFB* conditions. This result is unsurprising since it reflects an active re-adaptation toward baseline, rather than more spontaneous unlearning. More interestingly, however, we found that savings was stronger following washout than following *Clamp* and *NoFB* trials. Interestingly, the magnitude of the savings we observed following washout was weaker than we have observed previously in paradigms that used a smaller number of washout trials (Zarahn et al., 2008; Huang et al., 2011). Savings is likely dependent on the number of trials of washout (Krakauer et al., 2005). Here we used a relatively long washout block of 200 trials, compared to previous studies that employed only 80 trials (Zarahn et al., 2008; Huang et al., 2011). We suggest that this may have affected savings by increasing the value associated with baseline movements, rather than directly diminishing the value of the previously reinforced action at the end of adaptation.

We interpret the lack of savings in the *Clamp* and *No Feedback* groups as reflecting the fact the reinforced action has been completely erased. However, an alternative explanation is that the memory is indeed retained but subjects are unable to retrieve it due to interference caused by the multitude of movements made during the unlearning block that may have been equally reinforced. Indeed we have argued previously that interference is attributable to competition for retrieval rather than over-writing one memory by another (Krakauer et al., 2005). Although there was no direct reinforcement in the *No Feedback* group, subjects may have presumed that their movements would be successful, therefore receiving a comparable reinforcement and therefore giving rise to the same kind of interference.

We observed the greatest extent of savings in the *Time* condition. The Bayesian explanation for the faster re-learning following a period of inactivity is that uncertainty about the plant and perturbation increased during the idle period, so that new prediction errors had a relatively stronger influence on updating subjects' estimate of the perturbation (Körding et al., 2007; Wei and Körding, 2010). This logic should, however, apply equally to the condition in which visual feedback was removed. We found no evidence to support this theory in our data, however, since the learning rate during re-learning was identical when visual feedback was removed, compared with when feedback was clamped at zero error. We therefore favor the idea that savings was maximal after a period of inactivity because there were fewer (zero) intervening washout trials to reinforce baseline.

In summary, our findings, in conjunction with our previous work and that by others, lead us to conclude that spontaneous unlearning reflects reversion to baseline actions (which have presumably been strongly reinforced throughout life) from a new action that has been more weakly reinforced during adaptation. The presence of savings implies that the adapted action is not entirely forgotten. Thus, a weakly reinforced action can either be out-competed but not forgotten (*Time* and *Washout*) or out-competed and forgotten (*Clamp* and *No Feedback*). Future work will need to establish the degree to which our findings generalize to other motor learning paradigms, such as force field adaptation during reaching (Pekny et al., 2011) or split-belt adaptation of locomotion (Reisman et al., 2005), and to further clarify the interaction between internal models, presumably in the cerebellum, with a reinforced controller, presumably in motor cortex.

### Conflict of interest statement

The authors declare that the research was conducted in the absence of any commercial or financial relationships that could be construed as a potential conflict of interest.
